# “How many times did I not want to live a life because of him”: the complex connections between child sexual abuse, disclosure, and self-injurious thoughts and behaviors

**DOI:** 10.1186/s40479-020-00142-6

**Published:** 2021-01-04

**Authors:** Delphine Collin-Vézina, Mireille De La Sablonnière-Griffin, Marudan Sivagurunathan, Rusan Lateef, Ramona Alaggia, Rosaleen McElvaney, Megan Simpson

**Affiliations:** 1 McGill University, 3506 University St., Room 321B, Montreal, QC H3A2A7 Canada; 2 Université du Québec à Chicoutimi, Chicoutimi, Canada; 3 University of Western Ontario, London, Canada; 4 University of Toronto, Toronto, Canada; 5 Dublin City University, Dublin, Ireland; 6 Carleton University, Ottawa, Canada

**Keywords:** Child sexual abuse, Disclosure, Non-suicidal and suicidal self-injurious thoughts and behaviors

## Abstract

**Background:**

Meta-analyses have confirmed an association between child sexual abuse (CSA) and non-suicidal and suicidal self-injurious thoughts and behaviors (SITB), yet the mechanisms linking these factors are, to date, poorly understood. The goal of the current study is to explore one potential influencing factor acting in the association between CSA and SITB, which is the disclosure experience. Disclosure has been identified as a prominent factor in the healing process of survivors, with a lack of support following disclosures heightening negative outcomes. Exploring the impact of CSA disclosure on SITB is necessary to build effective prevention and intervention strategies.

**Methods:**

This qualitative study is part of a larger initiative spanning diverse research sites in Canada and in Ireland and aiming to lend voice to young people who were sexually abused in childhood/adolescence. Participants were recruited from community-based sexual abuse/assault agencies, hospital-based specialized clinics and child advocacy centres. The Long Interview Method, based on a branch of phenomenology, was used to guide research design and data collection. The current thematic analysis, informed by a stress-diathesis model, is based on a sample comprised of 21 ethnically diverse youth aged 15 to 25 who self-reported a sexual abuse experience in their childhood or teenage years and who, as part of the interview on their disclosure processes, revealed past or current SITB.

**Results:**

The thematic analysis led to the identification of four main themes that both confirmed past research and conceptual models on SITB, and provided new insights. Participants perceived a clear link between their CSA experience and SITB and other mental health issues. They offered their views on the meanings of SITB for CSA victims: to cope with abuse; to end the abuse; to express self-hatred and loneliness; and to let people know about their suffering. They described how negative disclosure experiences led to more nonsuicidal and suicidal SITB. Yet, participants also revealed that receiving support for their SITB created opportunities for CSA disclosure and support.

**Conclusions:**

This study showed complex connections between CSA experiences, disclosure and nonsuicidal and suicidal SITB. Understanding the reciprocal influences between SITB, CSA disclosure and help-seeking could better equip mental health professionals and caregivers to provide support and foster healing and recovery in CSA victims.

## Background

Sexual violence towards children and youth is a worldwide public health issue. Global trends drawn from three meta-analyses [1–3] indicate that about 1 out of 8 people experience child sexual abuse (CSA). This widespread phenomenon is more frequently reported by women, with these meta-analyses estimating prevalence rates ranging from 15 to 20%. Sexual violence experienced by boys and men, reported at about 10%, has long been underestimated and is gaining growing social recognition.

CSA can have profound and long-lasting negative impacts on victims relating to their physical and mental health, social well-being, and other life domains [4–8]. A recent umbrella review [6] examined findings from meta-analyses on 28 different long-term outcomes possibly linked to CSA, across three broad domains: adult psychiatric disorders, negative psychosocial outcomes and physical health conditions. Twenty-six of these outcomes were associated with CSA, among which were suicidal and non-suicidal self-injurious thoughts and behaviors (SITB).

SITB are defined as “thoughts and behaviors that entail imagined or actual intentional physical injury to one’s body and extend to more passive desires, such as wishing one were dead” ([9] p. 246). SITB can be suicidal, involving at least some intent to die. Suicidal ideation, which are “thoughts about engaging in a behavior to end one’s life” ([9] p. 246), and suicide attempt, “engaging in a potentially harmful or lethal behavior with some intention of dying from the behavior” ([9] p. 246) are included in the concept, suicidal SITB. Non-suicidal SITB does not involve an intention to die. These include non-suicidal self-injury, defined as the “direct, deliberate destruction of one’s own body tissue in the absence of suicidal intent” ([10] p. 9). Thus, suicidal ideation and thoughts, suicide attempts, and non-suicidal self-injury are all forms of SITB.

Estimates of increased odds ratio in sexually abused populations in recent meta-analyses [11–14] varied between 1.36 for suicidal thoughts and behaviors [13] and 3.17 when only suicide attempts were considered [11]. With regard to non-suicidal SITB, a recent meta-analysis [15] estimated the increased odds ratio among sexually abused individuals at 2.65. Although past studies have commonly used cross-sectional, retrospective designs and high-risk/clinical samples, (e.g. [16, 17]) increased odds of suicidal SITB in sexually abused individuals were also found in meta-analyses focused exclusively on longitudinal and twin studies controlling for a range of confounders ([12]; OR: 2.43) and in subanalyses on longitudinal [1.65] or populational [1.93] studies [14]. These findings stemming from robust studies conducted across different populations, suggest that while child maltreatment is associated with an increased risk of attempting suicide, CSA in particular, has a direct impact on SITB [11, 18].

### Mechanisms linking CSA to SITB

Though not specific to SITB, Finkelhor and Browne’s [19] framework delineating the impact of CSA is an insightful framework for understanding the internal turmoil created by the abusive experience. Their traumagenic dynamic model identifies four dynamics of trauma that can manifest and alter cognitive and emotional functioning: traumatic sexualization, betrayal, powerlessness, and stigmatization. Traumatic sexualization refers to the impact on the child’s sexual development; betrayal represents the consequences of the breach of trust inherent in the experience of CSA; powerlessness reflects the disempowerment and lack of agency that results from the abusive experience; and stigmatization illustrates the internalized sense of badness, shame and guilt, common characteristics of those who have experienced CSA. CSA victims may struggle emotionally due to these four dynamics, manifest in psychopathology such as depression, post-traumatic stress disorder (PTSD), or eating disorders, a feature of which may be SITB.

SITB are never the consequence of one single cause or stressor, but rather arise out of several factors ranging from biological to psychological and sociological [20, 21]. Current theoretical conceptualizations of SITB are characterized by a multiplicity of competing theories [21]. Models drawing on a stress-diathesis framework have been repeatedly proposed; they posit that proximal and distal risks factors must interact to lead to suicidal SITB [20]. Distal risk factors are elements that predispose an individual to suicidal SITB, including genetic, cognitive and social vulnerabilities [22]. Proximal risks are those at play in the daily lives of individuals exhibiting suicidal SITB. Among the most documented proximal contributing factors to suicidal SITB are psychiatric disorders, including mood, substance-related, anxiety, psychotic, and personality disorders, as well as acute psychosocial crises [20]. Based on this conceptualization, CSA can thus act as a proximal factor – a crisis experience – through the mental health challenges that follow such traumatic experiences. One could also conceptualize the disclosure of CSA as an acute psychosocial crisis that may heighten risk of suicide ideation and attempt. CSA may also play a more distal role in suicide, through the cognitive and social vulnerabilities that follow CSA as per Finkelhor and Browne’s traumatogenic model.

Building upon the stress-diathesis hypothesis, the interpersonal model of suicide attempted to address the lack of differentiation, in previous models, between individuals with thoughts of suicide from those who attempted suicide [23, 24]. This model suggests that, in order for one to develop active desire for suicide, individuals need to experience thwarted belongingness (through social alienation, loneliness) and must perceive burdensomeness (feeling like they are a burden to others, self-hatred). While experiencing either can lead to suicidal ideation, it is their combination that creates hopelessness leading to active desire for suicide. For this desire to move to suicidal intent and attempt, individuals need to acquire suicide capability. This capability is contingent on some genetic vulnerabilities and on experiencing repeated exposure to painful, provocative events, including childhood abuse [24] and non-suicidal SITB [25]. This model enables a more thorough understanding of how not only the actual experience of CSA, but also its consequences, may bring individuals who have experienced CSA to suicidal ideations and attempts.

Although not a direct determinant of suicide, previous non-suicidal SITB are considered a severe risk factor for suicide [20], and have been posited as one means through which individuals acquire suicidal capability [25]. Non-suicidal self-injury can serve several intrapersonal (e.g., affect regulation) and interpersonal (e.g., help-seeking) functions [26]. Indeed, adolescents who struggle to express their emotions and inner experiences have increased depressive symptoms [27] and thoughts of suicide [28]. This functional model has been integrated within a stress-diathesis approach [23]. Distal risk factors, including child maltreatment, are posited to create intrapersonal and/or interpersonal vulnerabilities associated with engaging in non-suicidal self-injury. In addition, this model proposes that vulnerabilities specific to non-suicidal self-injury interact with the stress component in order to lead an individual to non-suicidal self-injury specifically. The six hypotheses proposed as creating non-suicidal self-injury specific vulnerabilities draw on previous research and are: social learning, self-punishment, social signaling, pragmatic, pain analgesia, and implicit identification. For example, the social-signaling hypothesis argues that, after attempted coping strategies have failed to achieve the desired outcome, such as speaking to others about one’s feelings, some individuals may turn to a higher intensity social signaling, such as non-suicidal self-injury, in order to be heard [29].

Expanding on this work, the benefits and barriers model of non-suicidal self-injury highlighted five barriers and four benefits associated with these behaviors [30]. The five barriers are: a lack of awareness about non-suicidal self-injury, a positive view of the self, physical pain, aversion to non-suicidal self-injury stimuli, and social norms.

These barriers need to be lowered for an individual to engage in non-suicidal self-injury and access its benefits, which are self-punishment, affective, affiliation, and communication. The affective benefit is associated with all non-suicidal self-injury events, and childhood abuse is specifically conceptualized as a distal risk factor for the self-punishment and the communication benefits. In addition to representing a cry for help, victims of CSA may thus engage in self-injury as a response to intolerable events that they have experienced. Victims may self-harm in an attempt to manage or reduce severe distress or anxiety in search of relief from emotional pain; to express internal feelings of guilt and shame in an external manner via self-punishment; and to communicate their depression or distressful feelings to others.

Despite the strong association established between CSA and SITB in past research, the mechanisms linking these factors are yet to be fully understood. The goal of the current study is to explore one potential influencing factor in the association between CSA and SITB, which is the disclosure experience.

### Child sexual abuse disclosure

Disclosure of traumatic events is a very complex and iterative life-long process [31]. Despite recent social movements and prevention campaigns that encourage CSA survivors to disclose their abuse and seek professional help, victims often delay reporting or never tell [32]. The premise is that the sooner a victim of CSA discloses their abuse, the sooner they will be able to receive professional and social support, and this may lessen the risk of the many possible repercussions of CSA. The assumption is that survivors who disclose will be met with empathy and support; however, this is not always the case and recent studies have found mixed responses to CSA disclosures. For instance, among a sample of 487 male survivors of CSA who disclosed the abuse in childhood, approximately 57% reported being believed by their mothers, yet only 29% felt supported [33]. Among the same sample, 79% of survivors who disclosed to a person other than their mother were believed and 34% felt they were supported. Other studies have also found mixed CSA disclosure responses among both female and male survivors [34], even when disclosing to health professionals whom survivors expected would be able to support them [35, 36].

Although the relationship between support following disclosure and children’s post-disclosure functioning is unclear [37], disclosure is nonetheless a prominent factor in the healing process of survivors and has been described as the first step to recovery from CSA [38]. Most studies on the impacts of disclosure on the wellbeing of CSA survivors have examined the types of disclosure responses received – positive or negative. Positive responses to CSA disclosures have been found to result in feelings of relief, reassurance, and healing [35], as well as fewer psychological symptoms [39]. Notably, one study found that the greater the number of individuals to whom the sexual abuse was disclosed, the lower the amount of somatic complaints and aggressive and intrusive behaviours was evident among these CSA survivors [40]. Negative responses to CSA disclosures have been associated with sexualized behaviour among children [41], sexual revictimization in adulthood [42], and poor coping strategies [43, 44]. A lack of support following disclosures can hinder the recovery process [34] and increase distressing feelings, such as shame, that can exacerbate the association between CSA and STIB [45].

Considering the important role that disclosure has in the recovery process of CSA survivors and its potential to contribute to positive or negative sequelae, the outcomes of CSA disclosures continues to warrant scholarly attention. Whereas most studies on disclosure have focused on initial responses to disclosures and the impact of this on the individual victim, less research has investigated harmful behaviours throughout the disclosure process. One such area in which the literature is scarce concerns the issue of suicidal and non-suicidal SITB of survivors. In a study of women under 25 years of age who engaged in non-fatal suicidal behavior, Curtis [46] found that many of the participants who reported a CSA history described negative CSA disclosure experiences; however, this author noted the absence of research on the relationship between sexual abuse disclosures and suicidal behaviour. There is some evidence to suggest that adult CSA survivors’ perceived support from family and friends (not specifically related to disclosure) buffered the effects of CSA on the risk of suicidal ideation [47]. Therefore, it could be speculated from these findings that positive, supportive disclosures may have the potential to decrease the risk of self-injury and suicidality among CSA survivors. Considering the prevalence of SITB among CSA survivors, and the scarcity of studies investigating the impact of disclosures on such behaviours and thoughts, exploring the impact of CSA disclosure on SITB, during and after the disclosure, is necessary to build effective prevention and intervention strategies.

## Methods

This qualitative study of youth, who were sexually abused in childhood/adolescence spanned diverse research sites in Canada (Quebec and Ontario) and in Ireland. The study was designed using a consensual qualitative research approach (CQR) [48], which emerged out of phenomenology [49] and grounded theory methodology [50]. CQR aims to capture inner experiences and to develop conceptual frameworks that challenge normative assumptions, based on in-depth, lived experiences of participants. CQR is particularly well-suited to the objectives pursued in the current study as it values collaboration of a team to capture the complexity of the data. It is “based on the assumption that complex issues involve multiple perspectives and levels of awareness” ([48] p. 523). CQR assumes that research bias is less likely when several researchers are involved.

Using a CQR approach, the Long Interview Method [51] was chosen as the specific method to collect data. This is an inductive, discovery oriented qualitative design, compatible for collecting data involving process issues and for uncovering complex processes. The Long Interview Method has a well-established semi-structured in-depth interview framework for collecting data. The interview guide for this study was developed with this framework in mind, consisting of demographic questions followed by a series of question areas which are outlined in the data collection section. As with other phenomenological methods, it aims to obtain embodied, experiential meanings to rich descriptions of a phenomenon or human experience as it has been experienced [52, 53]. This method lends voice to the ‘lived experience’ of CSA survivors.

Using a CQR approach, the Long Interview Method [51] was chosen as the specific method to collect data. The Long Interview Method is a well-established semi-structured in-depth interview approach in qualitative research. Like other qualitative phenomenology methods, it aims to obtain embodied, experiential meanings to rich descriptions of a phenomenon or human experience as it has been experienced [52, 53].

### Sampling

Participants were recruited from community-based sexual abuse/assault agencies, hospital-based specialized clinics and a child advocacy centre. The recruitment agencies were located in urban or semi-urban settings. These young people were interviewed between November 2015 and June 2018. The study advertised for individuals currently aged 14 to 25 who self-identified as having experienced sexual abuse during childhood/ adolescence (e.g. before the age of 18). All participants were either currently receiving or had recently received services for their CSA experiences. This requirement ensured that no participants were divulging their experience of CSA for the first time through the study and that, if needed, appropriate service providers were informed of the situation for response. Participants were recruited through posters at agencies (e.g. waiting room, bathrooms). Staff in the participating agencies were also instructed to give recruitment brochures to eligible potential participants (youth aged 14 to 25 who had experienced sexual abuse before the age of 18). Participation in this research was voluntary and had no impact on access to services provided by the agencies. Youth over the age of consent according to each site’s legislation and/or research guidelines gave informed written consent; the age of consent for this research was 14 in Quebec, 16 in Ontario and 18 in Ireland. For the youth under the age of consent, parental consent and the youths’ assent were obtained. Ethics approval was obtained from the research ethics boards of the three universities, and agencies that had their own internal ethics board. Written consent/assent was gained prior to conducting the interviews; with participants who chose a phone interview, consent was also obtained verbally and was digitally recorded.

The study is based on a larger disclosure study involving 47 participants, 21 (44.7%) of whom referred to suicide ideation, behaviours or attempts, or self-injury in their interviews. Among those 21 participants, two participants identified as males and 19 as females. The average age of those 21 participants was 19 years old, with the youngest participant being 15 years old and the oldest being 25 years old. Participants were asked about their identified ethnicity, country of birth, and cultural group, and the responses demonstrated the diversity of participants in this study. Cultural identities reported by the 21 participants included Hispanic backgrounds, Croatian, Canadian, Québécois, Caucasian, Irish, Jamaican, Scottish, Polish, Portuguese, and Tajikistan. Eighteen of the participants were born in Canada or Ireland.

Most participants (*n* = 18) reported an experience of CSA by one perpetrator, with varying duration from a single event to multiple abusive experiences occurring over many years. One participant experienced both extra and intra-familial CSA, and two participants reported two or three different CSA experiences (either extra or intra) that involved different perpetrators. Fourteen of the participants experienced intra-familial CSA and the perpetrators included grandfather (*n* = 1), the mother’s partner (non-biological father; *n* = 2), fathers (*n* = 4), brother (*n* = 1), cousins (*n* = 4), and uncles (biological and through marriage; *n* = 2). Ten of the participants experienced extra-familial CSA and perpetrators included family friends (*n* = 3), online predator (*n* = 1), a stranger, friends of a sibling or peer (*n* = 3), boyfriend (*n* = 1), friend (*n* = 1), and a cousin’s partner (*n* = 1). Of the perpetrators of these 24 CSA experiences, only one was a female perpetrator.

Among the 24 CSA experiences endured by these 21 participants, only one experience was disclosed less than a year after the events, and four participants disclosed 1 to 2 years post-CSA. Thus, the majority of the 21 participants (*n* = 16) reported a delay in disclosure of several years. The longest delay from CSA onset to disclosure was 15 years for one participant.

### Data collection

An interview guide was crafted based on previous work with CSA survivors [54–57], with special attention given to reaching consensus on a schedule that allowed for implementation across sites. This covered the following broad topics: how and in what ways they disclosed CSA; what facilitators and/or barriers to formal and informal disclosures of CSA and other forms of victimization did they identify; outcomes of disclosures; interactions with service systems (i.e. legal professionals, counselling services school personnel) involved upon disclosure; advice to other youth; and recommendations for service providers. The average length of the interviews was one hour and seventeen minutes each. One interviewer conducted the interviews in Quebec (Canada), two interviewers conducted interviews in Ontario (Canada) and three interviewers conducted interviews in Ireland.

In Quebec, the interviews were conducted in the preferred language of the participant (French or English). To ensure that all co-researchers had access to the data collected, the verbatim transcriptions of the interviews conducted in French were translated by a certified translator and reviewed by the bilingual research assistant who conducted the interview and the bilingual research coordinator. Finally, additional resources were provided to participants if needed, and interviewers made one follow-up phone call a week after the interview to ensure participants were not experiencing upsetting feelings following the interview.

### Data analysis

The Long Interview Method is highly compatible to a thematic analysis. Data analysis of the 21 CSA transcripts that included experiences of self-injury and suicidality was carried out using a specific form of thematic analysis (reflexive thematic analysis) as outlined by Braun and Clarke [58]. Transcribed interviews were imported into a data analysis software program, NVivo [59], and coded for theme extraction and in-depth interpretation. The codes were generated in an inductive manner, with the codes identified from the transcripts. Similar codes were collated into coherent clusters or substantial codes being “promoted” to emerging themes.

Trustworthiness was established through the investigators’ long involvement in this field of study, persistent observation of the data, and provision of quotes for confirmability of themes [60]. Following the CQR approach, the research team across the three sites met to review the data, share observations and preliminary interpretations and to revise and define final themes. Intermittent meetings were held using Skype and Zoom to discuss and determine themes.

## Results

The thematic analysis led to the identification of four themes: 1) Linking CSA to SITB and other mental health issues; 2) Understanding the meanings of SITB for CSA victims; 3) Experiencing the negative interaction between disclosure and SITB; and 4) Seizing opportunities for disclosure and support through SITB manifestations.

### Linking CSA to SITB and other mental health issues

The 21 participants who reported non-suicidal and suicidal SITB during the interview unanimously perceived these symptoms as intrinsically linked to their CSA experiences. They talked about SITB manifestations as directly connected, and even attributable to CSA.

*All of those years was for nothing, for him to do this. How many times have I wanted to kill myself. How many times did I not want to be alive because of him (the abuser).* (Female, 18)

*I mean, like, there were definitely moments (during the abuse) where I did wanna like end my life and whatnot.* (Female, 15)

*I was dealing with a lot of suicidal thoughts, um, which wasn’t new, suicide stuff, I mean, the, the want to an actual killing of myself was new but the thought of death was pretty much prevalent throughout my childhood (during the abuse).* (Female, 21).

They also discussed how these behaviors took part in the course of a global mental breakdown associated with CSA, and that their overall mental health status was very fragile.

*I was suffering really bad with my mental health. I was really suicidal* (Female, 24)

*Last year was very, was very bad for me. Um I … I had a lot of suicidal thoughts. I had a lot of um, like my mental state was very all over the place.* (Female, 17)

These mental health issues were, for some participants, more directly linked to internalized problems, including depression, anxiety and eating disorders.

*I basically kinda came out about the fact I had really bad depression and I was self-harming and I was suicidal and all that, so they were trying to keep me on track basically cause it was almost like as if they could see I wasn’t doing well. It was like you could look at me and I could break*. (Female, 21)

*Coming from like the depression, anxiety, uh, the self-harming. I-I had an eating disorder like, not only was it the abuse coming from my father and what he told me, what, how he tried to mould me into the perfect girl, under his eyes, if you want.* (Female, 16)

Other participants described this mental health breakdown as involving externalized problems, including anger, substance abuse, and acting-out.

*I was just constantly angry at everyone and I went through bouts of self-harming and being a risk to myself and I wasn’t very well adjusted. I couldn’t really cope with anything, I was freaking out over little things, I was wanting to leave school, I didn’t … So I wasn’t – I wasn’t good.* (Female, 21)

*I did self-harm, you know, I did overdose on a few, medication here and there. I’ve been to hospitals, I’ve been to doctors.* (Female 19)

*And they came in and unlocked the house and unlocked the bedroom door and I was there in bed. I was covered in sick, I was covered in blood, I was just in a really bad way. I had self-harmed, I had overdosed, I had just taken pills and gotten sick and I just lay there, just lay in that bed for a week, couldn’t pull myself out of it.* (Female, 21)

*Like it got to the point where I was, l like I tried to commit suicide a bunch of times, I started getting into legal trouble, I started running away* (Female, 18).

### Understanding SITB for CSA victims

Participants in this study described the multifaceted meanings behind their SITB manifestations. They attributed four main rationales for these thoughts and behaviors: to cope with abuse; to end the abuse; to express self-hatred and loneliness; and to let people know about their suffering.

The first rationale was as a coping strategy. Many participants described how SITB were used as a means of dealing with intolerable experiences, that it helped them to escape and avoid the painful feelings associated with the CSA.

*I was self-harming during the time of the abuse. I just found it a way to cope with it.* (Female, 15)

*It was like frenetic because the thing is like, the self-mutilation helped me a lot with … uh … all that stuff.* (Female, 23)

*Lot of escapism (to cope) and then eventually, as I matured that translated into suicide, so suicidal thoughts* (Female, 21).

Linked to the use of coping strategies but more directly connected to ending their lives, the second rationale was as a way to end the abuse. They described suicide as a way out, as a means to end the suffering they were enduring due to the sexual violence committed against them.

*To just end my life … And the thing was I was very vulnerable ( … ) Like there was one time I was really suicidal I was happy about the thought of killing myself. I remember one night being just being so calm and ah getting something sharp out of the kitchen drawer and going off to my bedroom but I think a friend text me or something but I ended up not doing much damage like you know I think I cut my arms but that’s about it and my stomach, even it was like as if I was finally going to come to terms with it, finally be at peace but I’m still here.* (Female, 21)

*I attempted suicide three times in fact between the ages of thirteen and sixteen ( … ) The experience for me, well it’s that I was tired then of living that, uh, it’s basically my father ( … ). By killing myself, it was my only way. ( … ) It’s, my goal of dying, was to never see him again y’know.* (Female, 19).

The third rationale that emerged for SITB was as an expression of self-hatred and loneliness. They described feeling bad, dirty and estranged from others, and how these feelings were interconnected with their SITB behaviors.

*And … um … well one day I decided that, I was dirty and that it was a good idea to [silence] it was a good idea to, [voice breaks, sigh] ‘it’s very strange it seems like a cult’ but to purify myself ( … ) but it wasn’t a call for help I mean it wasn’t like, it was just a thing like, ‘Listen, I’m tired of having nightmares and feeling like shit*. (Female, 23)

*I used to self-harm and I use not to be able to talk to my friends, I used to think I was so different like, thought like I was just a weirdo*. (Female, 25)

*I didn’t have many- no more friends, I didn’t talk to anyone, and around the age of eleven, I started rebelling. I tried to kill myself several times*. (Female, 15)

The fourth rationale described was to let people know about their suffering. They described their multiple and often unsuccessful attempts to be seen and heard through their behaviors and how this acting-out was serving the function of a cry for help.

*A cry for help. I think it was a cry for help, that my body, that my- my head had had enough of that. And it was like a cry for help- for help, like an SOS, like someone help me.* (Female, 15)

So I went to her (a friend) anyway: “look at what am I doing. what am I doing.” (showing her cuts)*.*

(Female, 18)

*Well when I was thirteen I tried to commit suicide. Afterward they asked me why, I talked badly about my father (abuser), I said my father wasn’t OK um (...) Um, I self-, I mutilated myself, I said, I spoke out against my father, I wrote suicide letters where I wrote lots of things, where I spoke out against my father*. (Female, 19)

Some participants were purposefully making these attempts in order to be acknowledged by others, while others remained unclear as to whether their cry for help was intentional or not.

*When I was thirteen I attempted, I was self-mutilating. Also before the attempt, um, I was self-mutilating a lot, you could see it, and I was taking dance classes so they could see it y’know I was doing it on purpose*. (Female, 19)

*Yeah except that the thing is, I went too deep I don’t know if it was an unconscious or conscious decision, there came a time like, there came a time I was a zombie.* (Female, 23)

### Experiencing the negative interaction between disclosure and SITB

Several participants described the negative interplay between CSA and SITB, and more notably how disclosure experiences heightened their suffering and thus their acting-out behaviors. They revealed how their disclosure was experienced as a crisis that left them with an intense feeling of being exposed to others and unsupported.

*No, it was really bad when, like a few years ago when I first told and stuff like that and then it was really bad and I … . Yeah. It was like the build up. Like I thought, I started to become really suicidal like maybe in the September/October and then I told in the November. So it was like a build up kind of thing of it like and then after I told I was really, really suicidal. Because then everything’s coming to the surface. It’s not pushed down, it’s not in the back of your head, it’s right at the surface like and everyone knows.* (Female, 15)

*Until after a period of fairly tough summer, last summer (when she disclosed to the family), I had an unsuccessful suicide attempt in September.* (Female, 24)

One of the participants clearly mentioned how she started engaging in self-injuring behaviors due to the pressure she felt from her friend to tell her parents about the abuse.

*(my friend said) if you don't tell your mom (about the abuse) I will. because I was breaking down to her, in that, I don't know what to do. ammm, so she, I had cut myself at the time.* (Female, 18)

Many participants in this study explained how unsuccessful support following disclosure led them to engage in even more self-injuring and suicide behaviors. They mentioned experiences of feeling not understood, not being helped quickly enough, or not feeling safe in using strategies that services were recommending.

*She (child protection worker) told me “I think you weren’t comfortable anywhere.” After that, I attempted suicide because um, I said to myself, “Yeah, she’s right, I’m not comfortable anywhere.” I’d be better off dead.* (Female, 19)

*well we (the participant and their mother) told eh the.. we told eh, the social worker and it obviously went to the guards (police) and everything but then in the December, I was put on a waiting list for (a service) and in December I took an overdose and was in hospital and that obviously sped up my.. time to go to the referral.* (Female, 18)

*I’m gonna say I, I told the therapist that like I was feeling suicidal or something, and even though I was getting help for it, like she still told child protection services.* (Female, 15)

*(Child protection) said are you going to tough it out because I was living with my father, well not all the time, I also had, I was, I was living with (name of a female friend), one of my friends and well, they said to me “Are you going to tough it out until next week? We’re going to meet with your father.” I told them “Yes” but I knew that if they talked to my father about it, he was going to come back really angry y’know knowing that I talked, so I attempted suicide*. (Female, 19)

Overall, they also mentioned that even when services were helping, participants were walking a fragile healing path. They noted how problematic symptoms were not always decreasing after receiving support and that ‘up and down’ patterns or an unexpected increase in symptoms despite receiving services were part of their experience.

*I had started self-harming and tried to commit suicide … more than once, ammm, and I suppose, (the organization) was helping and then … the counsellor was like, oh I am happy with you. I thought I was in a good place, so we ended on good terms and then when I hit back into school it just hit me. just, I don’t' know if it was a combination of the stresses of exams and everything but I just went downhill. Within about 4 weeks. I started self-harming and having suicidal thoughts.* (Male, 19)

*After three weeks (of starting counseling), I was suicidal. Y’know? Which is- which is actually strange because everyone thought I was worse than before. Which was totally wrong. Before, y’know when I was completely catatonic and everything? It’s like, I was doing so badly that I couldn’t be suicidal.* (Female, 19)

### Seizing opportunities for disclosure and support through SITB manifestations

In sharp contrast with challenges faced with disclosure and increased symptoms of SITB, participants in the study also described these symptoms as acting as facilitators to disclosure and support. For some participants, becoming conscious of the link between CSA and SITB became a turning point that led them to decide to break free of the secret they were holding and led to the initial disclosure.

*I came to understand what had happened to me and felt that it was necessary to say something at this point with just how I was feeling. Just my thoughts of not wanting to be around, so, that wasn’t a nice feeling to feel as a young person. To not want to live. ( … ) It was a feeling of not wanting to be around, of wanting to end my life and remembering life prior to my mom remarrying this person. So remarrying my—both of our abusers. Um, {Pause} I remember happiness and joy and just a different way of life and I wanted that back—I didn’t fully understand why I was feeling the way I was feeling, like, I understood what had happened to me, I, there was a description for it, it was in the dictionary, like I could read it over and over again but still to me it wasn’t enough and it was just, it felt too overwhelming so I needed to say something and couldn’t keep it inside anymore and it just felt like I was going to just end it. So, I just, I needed to do something about it. It was just a breaking point for me.* (Female, 24)

*The other time was, I just, again I just didn’t go into school and I just walked up to the local railway bridge and just sat on it for a good 45 minutes. Just going, ‘jump or not jump?’ that kind of way and that kinda, that did frighten me. and then, about a week later was when I went to just walk about and then, when my mom and dad came to get me, I kind of broke down and told them all this.* (Male 19)

For many participants, the acting-out behaviors were a springboard to enable them speak out about the abuse and receive support from family members.

*I’ve stopped self-harming but I used to. So there was this time where, like, they knew that I used to self-harm [Right]. They knew about that and like, of course I wasn’t doing it but there was this one day where like I did it [Um Hmm] and then, they just like, they walked into me, like, you know, so then my uncle he got really scared cause I had, like, a panic attack [Um Hmm]. So then he, like, he, he helped me go, like, go through that process and then he sat down with me and then he gave me that talk [Um Hmm]. Like, he told me that, he’s like a father to me now [Um Hmm] and that he’s there, and that if I ever need anything he’s there.* (Female, 16)

*It’s when my mother discovered my cutting, my pills and then at one point I had no more crutches, so I was just like ‘Okay maybe I’m going to disclose this to my mother maybe that’s going to help me’* (Female, 23)

*So the GP (family doctor) was just like … he … no I went over to the GP actually before I said anything to my Mam and Dad and stuff, because I was really suicidal and I was like ‘I don’t want to be here’ and then I disclosed that (the abuse) and then we went back over to the GP and my Mam was like ‘my daughter is telling me that she was abused and that’s why.’* (Female 24)

Finally, participants also described how they felt seen and heard by counselors and therapists who were helping them with their suicidal and self-injury behaviors and to whom they decided to disclose the abuse.

*One of my friends found out. they actually seen the scars and they told the guidance counsellor of my school and then the guidance counsellor talked to me and rang my parents and then my parents asked me about it, and then they suggested (the organization) to me and I agreed to it.* (Female, 15)

*That was actually almost accidental I think because when I came back to the service here in 2016, I came in with suicidal thoughts and I had already been collecting pills and all sorts of things. And it was a lot of talk of what’s wrong and all that kind of thing. And then one day, it was a very kind of – yeah, accidental almost. Like I sat there, and I thought if I don’t say that that’s what’s happening, this isn’t going to go anywhere. Yeah, so then I said it and then I cried a lot. But it felt like a big relief actually. I felt like this can go somewhere now. Because I didn’t really have other things to work on. This is what’s been making me miserable, I thought. And I was like, if I don’t come out with this then I’m not going to get any better. So I did say it. But it was a very positive experience because I said it and I sat in silence for a while and then I started crying and the counsellor just said, “Wow, okay”, as in, “Oh, I hear what you’re saying”, and she kind of gave me the time to just process the fact that I’d just said it.* (Female, 21)

*Because I built such a relationship with trust. amm, I rang them from school one day, thinking that I was going to kill myself like, and I rang her (the counselor) and she took me. I went over. she was only across the road from my school. ammm, if I was at home, I could ring her and say I need to come in.* (Female, 18)

## Discussion

The goal of the current qualitative study was to explore a potential factor influencing the association between CSA and SITB, which is the disclosure experience. This study is novel, as it is the first to explore the interplay between suicidal and non-suicidal SITB and CSA disclosure experiences with attention given to perceived positive or negative outcomes. The sample was comprised of 21 ethnically diverse CSA survivors aged 15 to 25 who, as part of an interview about their disclosure processes, revealed past or current SITB experiences. This sample was largely comprised of self-identified female survivors and were recruited in both Canada and Ireland through community and clinical CSA services. The thematic analysis led to the identification of four main themes that both confirmed past research and conceptual models on SITB, and provided new insights: 1) Linking CSA to SITB and other mental health issue; 2) Understanding the meanings of SITB for CSA victims; 3) Experiencing the negative interaction between disclosure and SITB; and 4) Seizing opportunities for disclosure and support through SITB manifestations.

### Confirming the association between CSA to SITB and its perceived meanings

Building upon the stress-diathesis model [23] and confirming previous research on the link between CSA and SITB, the CSA victims in this study believed that their victimization was linked, if not responsible, for their non-suicidal and suicidal SITB. Participants perceived that CSA as contributing to their past and present mental fragility including the experience of both internalized and externalized symptomatology. This appeared as a common trend in the current sample of CSA survivors, in spite of their age range varying between 15 and 25 and their diverse ethnic backgrounds. The desire to harm oneself and/or experiencing mental challenges post-CSA is consistent with previous research that has concluded that CSA victims are likely to both internalize victimization experiences and display externalized behaviors such as anger, acting-out and substance abuse [61].

In addition, this paper elaborates on and provides rich support for enhancing our understanding of the nuanced motivations underpinning suicidal and non-suicidal SITB. More specifically, while four subthemes emerged, three were concerned directly with CSA victimization. Namely, participants described engaging in self-injury or attempted suicide to cope with the abuse, to end the sexual victimization, or to deal with self-hatred and loneliness associated with CSA. The fourth subtheme identified SITB as serving the function of a cry for help and letting people know about their suffering. These rationales or explanations are consistent with existing models on the functions or benefits served by non-suicidal SITB [26, 29, 30], more notably as means to signal/communicate one’s suffering, to deal with emotional turmoil, and to punish oneself. They appear to serve both intrapersonal and interpersonal functions, as laid out in these models of non-suicidal SITB. This is also consistent with Alaggia’s [54] model and Cossar and colleagues’ [62] findings that suggest that CSA survivors use behavioral gestures as an attempt to disclose their true experiences, to be seen and heard without taking the risk of speaking out directly about the abuse. The function described by participants as a means ‘to end the sexual victimization’ speaks to suicidal manifestations of SITB and complements existing models that describe childhood abuse as an intense crisis acting as a proximal factor influencing a person’s motivation to die [23].

### Exploring the complex relationships between CSA disclosure and SITB

Although the association between suicidal and non-suicidal SITB and CSA was well established in previous research, new insights about the perceived relationship with disclosure experiences, through the voices of victims, were highlighted in this study. This study documented both positive and negative interactions between disclosure and SITB. On the one hand, several participants revealed feeling caught in a cycle of negative disclosure experiences and subsequent SITB. Consistent with the stress-diathesis model [22], they mentioned how their disclosure was experienced as an acute crisis that triggered or increased their SITB. These disclosure experiences were described as creating pressure on them to further disclose, not unlike McElvaney et al.’s [57] ‘pressure cooker effect’, bringing all emotions to the surface and leaving them unsupported through the difficult disclosure journey. For some participants in the study, services were inadequate in meeting their needs following disclosure, which increased their distress and SITB. They also pointed out that disclosure is not a direct path nor a direct solution to reduce mental health symptoms. The recovery process was one characterized by challenges and various detours. On the other hand, numerous participants revealed that SITB initiated a disclosure path that led to receiving positive support. When participants were able to understand the connection between self-harming and their CSA victimization, or realized how severe their suffering was and how much of an impact CSA had on their lives, they took the opportunity to make an initial disclosure. This finding suggests that making sense of their own experiences was a key factor in facilitating a disclosure. For others, the suicidal and/or nonsuicidal SITB elicited a more supportive response from other people - family members and professionals alike - which in turn facilitated CSA disclosure. This is consistent with Nock and colleagues’ model highlighting the positive interpersonal function of SITB [26, 29] and Hooley and Franklin’s model about the communication benefits associated with SITB [30].

What seems to differentiate these negative and positive experiences of disclosure and SITB is the quality of support, understanding, and acceptance that these survivors perceived, in relation to both the disclosure of the abuse experience and the symptoms manifested. Support appears to reduce “thwarted belongingness and perceived burdensomeness” which is a key aspect in SITB models that focus on the interpersonal nature of these symptoms [24], and may also be crucial to reduce stressors that are playing out in the stress-diathesis model [23]. In other words, support seemed to play a dual role: it helped those who engaged in SITB to seize the opportunity to reveal the abuse and to feel heard and seen, while at the same time helping those who disclosed to reduce the risk of further engaging in nonsuicidal and suicidal SITB. These findings highlight a process, or dynamic, that presents disclosure and mental health issues as flowing and interacting, based on the influence of perceived reactions from others to both CSA and SITB. As such, disclosure experiences appear to be an important factor to consider in SITB process-based models, such as the one proposed by Hooley and Franklin [30] or Van Orden and colleagues [24]: as much as negative reactions from others can perpetuate cycles of SITB, positive and supportive relationships can act powerfully to break those loops.

### Implications for practice, policy and research

This study supports previous studies in suggesting that in addition to intervention and prevention efforts among general populations of children and youth (e.g. school-based prevention programs that promote a non-blaming discourse towards victims and recognize the multiple barriers at play in holding victims from disclosing), caregivers and professionals need support in gaining the necessary knowledge and skills to promote a positive response to youth victimization and mental health challenges [63]. The current study highlights the need to better equip those who are well positioned to provide support to foster healing and recovery in CSA victims (e.g. provide support and treatment necessary to empower victims, help them deal with trauma, and reduce risk factors that can impact on further mental health issues). This supports recent prevention initiatives that focus on parents’ participation as a fundamental element to successfully increase the acquisition of skills and knowledge in children and to facilitate children’s ability to disclose CSA [64].

This study also brings important leverage to revisiting social policies that guide actions taken with sexually abused children and youth. For example, including mandatory training on CSA to all professionals working with children and youth would strengthen their abilities to facilitate the disclosure of CSA (e.g. creating a safe place for children to talk), and to react appropriately when faced with a disclosure (e.g. having a reassuring and supportive attitude) [65]. Findings support the importance of screening for child abuse in services where young people present with self-injury and/or suicidal ideation/intent. Training for professionals should bring awareness about the impact of CSA and the likelihood that children may communicate their distress through different means, including SITB. This is particularly relevant in all mental health services where sexual abuse may not be the core focus of the intervention. The hidden and shameful nature of CSA makes it particularly difficult for young people to articulate their experiences of abuse, and acting-out behaviors may serve different functions that need to be acknowledged and addressed. Results from this study also provide insight for media campaigns to highlight the challenges that victims experience and offer solutions to overcoming these roadblocks to disclosure, rather than simply telling victims they should talk about the abuse.

### Limitations

This study has several limitations that are worth highlighting. First, the sample, which drew from a diverse group of youth and young adults, all self-identified as CSA victim or survivor, had already disclosed the abuse to at least one person, and were receiving community or hospital-based services. This recruitment strategy inevitably excluded the voices of those who have never disclosed the abuse or who have never sought treatment or intervention. We fully recognize that their experiences may be different from those expressed by the participants in the current study. Second, this qualitative study is also one that explored the perceptions of CSA victims, which cannot be used to establish causality in the associations noted by the participants. Third, most of the quotes did not allow us to distinguish fully between non-suicidal and suicidal thoughts and behaviors, and as such, more research is required to distinguigh the impact of CSA disclosure on lethal and non-lethal forms of SITB. Finally, the initial study, which was focused on disclosure processes, did not include specific questions about self-harm and suicide. Consequently, while 21 out of the 47 participants in the larger study spontaneously mentioned past or current SITB, more CSA victims may have reported these mental health challenges if prompted in the interview, which could have impacted the findings generated in the study.

## Conclusion

The current project, which aimed at lending voice to the lived experiences of young people disclosuring CSA, offers important insights to inform strategies to more effectively intervene with CSA victims and address their SITB manifestations. This research hopefully contributes to raising awareness of sexual abuse, reducing social stigma, reducing victim-blaming attitudes and behaviour, and results in a more compassionate discourse towards CSA victims in our communities. Findings from this study may demonstrate not only the challenges associated with talking about CSA, but also the potential benefits such as feeling supported and heard, working through the mental health challenges associated with the abuse, and finding a way out of the cycle of suffering and despair.

## Data Availability

The datasets used and analysed during the current study are available from the corresponding author on reasonable request. The availability of the dataset is also conditional upon the proposed project being fully approved by a recognized research ethics board.
